# Diffusion of curcumin in PLGA-based carriers for drug delivery: a molecular dynamics study

**DOI:** 10.1007/s00894-024-06023-x

**Published:** 2024-06-19

**Authors:** Alessandro De Giorgi, Francesco Maria Bellussi, Stefano Parlani, Andrea Lucisano, Emanuele Silvestri, Susmita Aryal, Sanghyo Park, Jaehong Key, Matteo Fasano

**Affiliations:** 1 Department of Energy, Politecnico di Torino, Corso Duca degli Abruzzi 24, Torino, 10129 Italy; 2 Department of Biomedical Engineering, Yonsei University, Wonju, 26493 Gangwon State Republic of Korea

**Keywords:** Diffusion, Polymeric carriers, Molecular dynamics, Water, Mass transfer

## Abstract

****Context**:**

The rapid growth and diversification of drug delivery systems have been significantly supported by advancements in micro- and nano-technologies, alongside the adoption of biodegradable polymeric materials like poly(lactic-co-glycolic acid) (PLGA) as microcarriers. These developments aim to reduce toxicity and enhance target specificity in drug delivery. The use of *in silico* methods, particularly molecular dynamics (MD) simulations, has emerged as a pivotal tool for predicting the dynamics of species within these systems. This approach aids in investigating drug delivery mechanisms, thereby reducing the costs associated with design and prototyping. In this study, we focus on elucidating the diffusion mechanisms in curcumin-loaded PLGA particles, which are critical for optimizing drug release and efficacy in therapeutic applications.

****Methods**:**

We utilized MD to explore the diffusion behavior of curcumin in PLGA drug delivery systems. The simulations, executed with GROMACS, modeled curcumin molecules in a representative volume element of PLGA chains and water, referencing molecular structures from the Protein Data Bank and employing the CHARMM force field. We generated PLGA chains of varying lengths using the Polymer Modeler tool and arranged them in a bulk-like environment with Packmol. The simulation protocol included steps for energy minimization, *T* and *p* equilibration, and calculation of the isotropic diffusion coefficient from the mean square displacement. The Taguchi method was applied to assess the effects of hydration level, PLGA chain length, and density on diffusion.

****Results**:**

Our results provide insight into the influence of PLGA chain length, hydration level, and polymer density on the diffusion coefficient of curcumin, offering a mechanistic understanding for the design of efficient drug delivery systems. The sensitivity analysis obtained through the Taguchi method identified hydration level and PLGA density as the most significant input parameters affecting curcumin diffusion, while the effect of PLGA chain length was negligible within the simulated range. We provided a regression equation capable to accurately fit MD results. The regression equation suggests that increases in hydration level and PLGA density result in a decrease in the diffusion coefficient.

**Supplementary Information:**

The online version contains supplementary material available at 10.1007/s00894-024-06023-x.

## Introduction

Curcumin, a yellow crystalline powder, is a polyphenolic compound extracted from the rhizome of the Curcuma longa [1]. Over the past years, it has drawn attention due to its diverse pharmacological activities like anti-carcinogenic, anti-inflammatory, antioxidant, antibacterial, and anti-proliferative [2–4]. However, the clinical use of curcumin is still limited due to its low solubility, low bioavailability, rapid metabolism, lower gastrointestinal absorption, and low permeability, which limit the therapeutic success of curcumin [5, 6]. In the quest to overcome the bioavailability issues and enhance its properties, better drug delivery systems have been explored.

With that purpose, carrier-mediated delivery of curcumin has emerged as a strategic approach. Research has been carried out to find efficient drug delivery systems able to enhance the poor solubility and retention time of curcumin in aqueous media [7, 8]. Several nanocarriers have been developed over the years, following different approaches [9–13]. However, only a few of them are clinically approved [14–16]. Among them, poly(lactic-co-glycolic acid) PLGA is attracting more attention due to its biocompatibility, biodegradability, and FDA approval [17, 18]. PLGA-based microparticles are widely used for the sustained release of drugs because they have several advantages over traditional pharmaceutical dosage forms [19–22]. The mechanism of drug release is usually directly related to the diffusion, swelling, erosion, and degradation of the polymer matrix [23]. It is generally accepted that PLGA is hydrolyzed by erosion to produce water and carbon dioxide from the intermediate lactic acid and glycolic acid monomers [24]. Another property of the PLGA polymers used to tailor drug release is their ability to hydrate. Therefore, changes in polymer water uptake lead to swelling and its eventual dissolution [25].

Nowadays sophisticated computational tools are available to design and optimize efficient nano and micro drug delivery systems, which otherwise require long and expensive experimental tests [26–29]. The intrinsic nature of micro/nano diffusion systems relies on different spatial and temporal scale phenomena, thus requiring the adoption of an approach able to span from the discrete to continuum domain [30, 31]. Numerous *in silico* investigations [32–36, 74] have been carried out to study and predict properties and biomolecular behaviors of PLGA at multiple scales. Some studies in this context [37, 38] have used a design of experiment (DOE) to highlight correlations between input parameters and their outputs, providing valid conclusions with minimal experimental tests, time, and cost. As an example, Iman Salahshoori et al. [37] used a DOE coupled to molecular dynamics (MD) simulations to study the effect of silica nanoparticle loadings, temperature, and pressure on the transport properties of $CO_2$, $CH_4$, and $N_2$ gases in a PSF-PEG-silica mixed matrix membrane.

However, the relation between curcumin diffusion through hydrated PLGA and the physical-chemical features of this system has never been assessed with molecular precision, despite being the fundamental step of a multi-scale method to assist the development of PLGA-based drug delivery systems. In this work, we combined MD simulations with the creation of a DOE [39], and a sensitivity analysis [40] to highlight important relations between the parameters investigated — namely the length of the PLGA chain (*L*), the hydration level (*H*) of the system, and the PLGA density (*P*) – and the diffusion of curcumin in a representative volume element of PLGA-based drug delivery systems. In perspective, the present investigation could lead to a more rational design of curcumin release from Discoidal Polymeric Particles (DPPs) made of PLGA. Beyond the specific study case addressed here, we aim to propose a structured computational protocol to assist the design of drug release systems.

## Materials and methods

### Molecular dynamics simulations

**Fig. 1:**
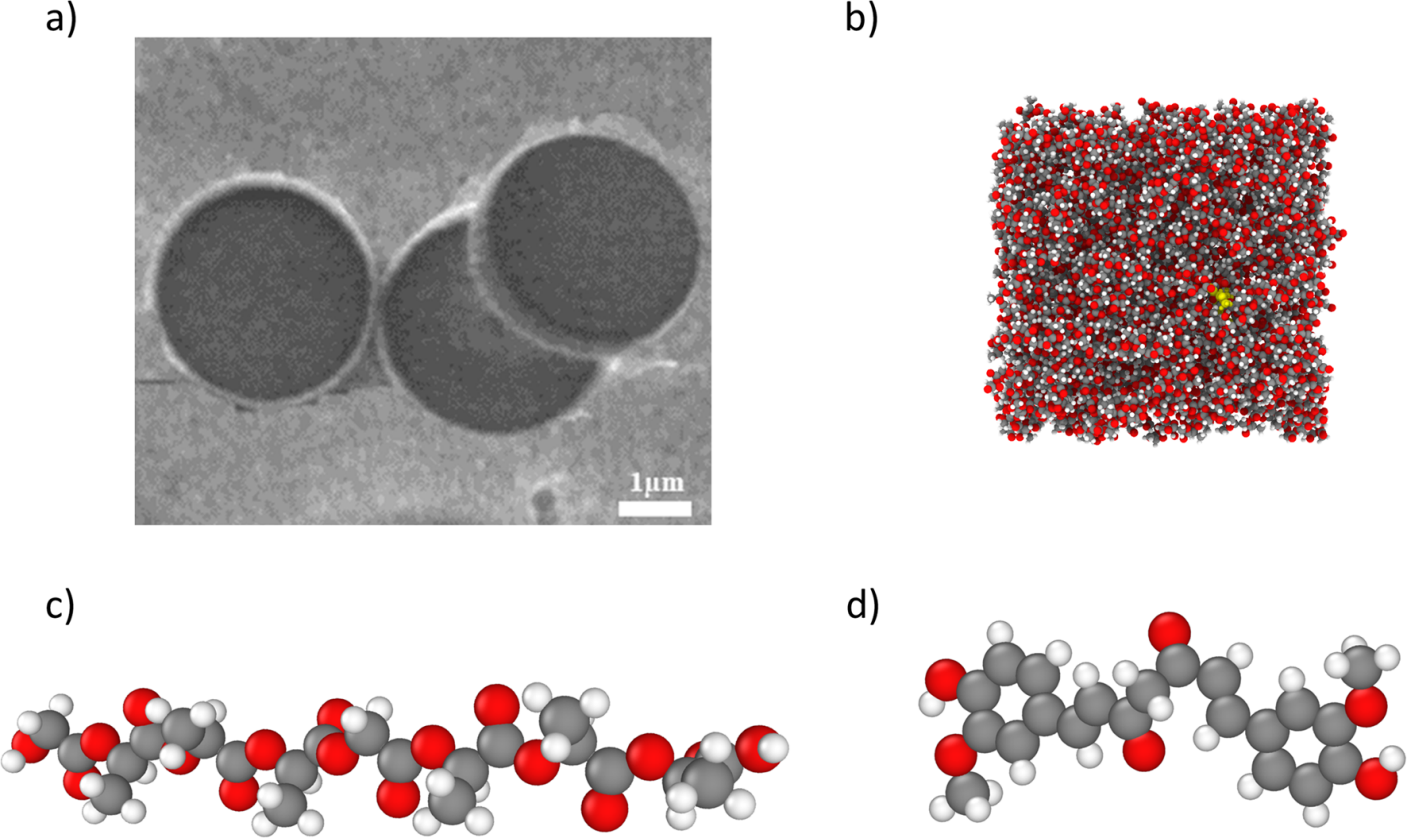
**a** Morphology of curcumin-loaded DPPs using Scanning Electron Microscope; **b** rendering of a simulated PLGA box, filled with curcumin (in yellow) and water; **c** rendering of a single chain of PLGA; **d** rendering of a single molecule of curcumin. Colors in **c** and **d** indicate different atoms type: carbon in grey, oxygen in red, and hydrogen in white

The computational system was created to mimic a representative volume element of curcumin-loaded PLGA DPPs (see Fig. 1a) [41, 42]. Thus, the simulated system consisted of a curcumin molecule immersed in a box composed by PLGA chains and filled with water (see Fig. 1b). MD simulations were performed in GROMACS. The topology of poly-lactic acid (PLA) and poly-glycolic acid (PGA), as well as curcumin molecules (see Fig. 1c and d), were obtained from Protein Data Bank [43–45]. The PLA and PGA monomers were linked to create PLGA copolymer chains using the Polymer Modeler tool on nanoHUB [46]. The chains were generated with a random distribution of monomer residues. Three types of PLGA chains were examined, each with different lactic:glycolic molar ratios and varying lengths: 1.8 nm (0.40:0.60), 5.0 nm (0.46:0.54), and 14.7 nm (0.50:0.50).

**Fig. 2:**
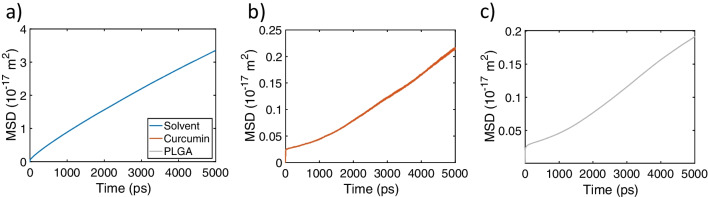
Exemplified mean square displacement (MSD) of **a** solvent, **b** curcumin, and **c** PLGA

The single chains were then packed to achieve a larger computational domain of bulk PLGA using the software Packmol [47]. According to the different PLGA chain lengths and densities, up to 60 chains were included in the computational domain. The CHARMM force field was considered [48], one of the most adopted force fields in biomolecular and drug design studies [49]. Moreover, the CHARMM force field is already parametrized both for curcumin and PLGA without requiring further fundamental computational iterations, such as *ab initio* or density functional theory simulations, to obtain the necessary parameters for molecular dynamics simulations. CHARMM parameters were downloaded from SwissParam [50]. Nonbonded interactions with up to 1.2 nm cut-off radius were considered, being long-range electrostatic interactions computed with the PME method [51] ($10^{-4}$ accuracy, 0.4 nm spacing grid). The SPC/E [52] model was considered for the water molecules. The number of water molecules added to each configuration depended on the hydration level set for each simulation (see Tables S3 and S4 for information about the box size and number of water molecules in each simulation).

For the preliminary energy minimization step, the steepest descent algorithm was used. The system was then equilibrated at 300 K considering several NVT and NPT simulations (time constant for temperature and pressure coupling equal to $\tau _T=0.2$ ps and $\tau _P=3$ ps, respectively) to achieve the target PLGA/water density. Temperature and pressure equilibration was carried until steady state, with runs lasting 4 ns or more. Finally, the production run was carried out considering an NVT ensemble with Nosè-Hoover thermal coupling. This thermostat (target temperature: 300 K; time constant coupling: 0.2 ps) was applied to the three groups of atoms that build up the system (water, PLGA, curcumin) to avoid any hot solvent-cold solute issue [53]. Production runs lasted 5 ns to have enough statistics to extrapolate results.

The isotropic diffusion coefficient *D* was evaluated considering the mean square displacement (MSD) of individual atoms of each species in the system as a function of the observation time using the classical Einstein relation for Brownian motion [54]:

1 $$\begin{aligned} MSD=\lim _{t\rightarrow \infty } < \parallel \mathbf {x_{i}}(t)-\mathbf {x_{i}}(0) \parallel ^{2}>=6Dt . \end{aligned}$$

MSD data were computed along trajectories of 5 ns for each species of the system (see, for instance, Fig. 2a, b, and c). A linear fit was performed on the MSD over time. For each simulation, a linear region was identified (see Supplementary Figs. S1-S6 for MSD and Tables S1-S2 for the linear fitting time intervals considered for all configurations), and diffusion coefficients were derived from these linear sections. Additionally, a test simulation from DOE1 was extended to 10 ns to check system convergence and autocorrelation: the percentage relative errors between the 10 ns and 5 ns simulations were then computed for each species, yielding a limited discrepancy of 4.82% for water, 2.14% for curcumin, and 0.42% for PLGA in terms of *D* values (see Supplementary Fig. S7). The Tukey’s fences inspection [55] was adopted to eliminate possible outliers among the acquired data (see Supplementary Note 1).

### Design of experiment

A design of experiment (DOE) approach was followed to gain deeper insights and correlations between the input parameters and the corresponding outputs of the simulations. A combination of sensitivity analysis (SA) with the DOE technique generally helps to obtain the most reliable results in this context. The chosen structure of the DOE was the Taguchi method [56], which is a simplified alternative to a full-factorial method (FFM) [57]. More information regarding the Taguchi method and how it was adopted in these MD simulations can be found in Supplementary Note 2. The input parameters selected for the DOE included hydration level (*H*), PLGA chain length (*L*), and PLGA density (*P*).

In a first set of numerical experiments (DOE1), three different levels were considered for each parameter, namely: $L=$1.8 nm, 5.0 nm, and 14.7 nm; $H=$0.600 g/cm^3^, 0.800 g/cm^3^, and 1.000 g/cm^3^; $P=$0.804 g/cm^3^, 1.072 g/cm^3^, and 1.340 g/cm^3^. Notice that the upper bound of *L* was determined on the basis of the maximum box size that could be simulated with the available computational facilities; the highest value of *H* was the bulk density of water at the simulated thermodynamic conditions, meaning capillary condensation and thus full hydration of the PLGA carrier; the upper bound of *P* was considered as the bulk density value of PLGA — lower values could be possibly achieved when swelling occurs. Hence, the L9 Taguchi orthogonal matrix was designed, providing the 9 configurations to be simulated (see Table 1). To better investigate the behavior of the system in a broader range of hydration levels — which showed to be an important factor determining *D* — a second set of experiments (DOE2) was performed, considering the same parameter levels of DOE1 but $H=$ 0.000 g/cm^3^, 0.300 g/cm^3^, and 0.600 g/cm^3^ (see Table 2).

**Table 1:** Taguchi orthogonal matrix for the DOE1, where *L* indicates the PLGA chain length, *H* the hydration level, and *P* the PLGA density

	*L* (nm)	*H* (g/cm^3^)	*P* (g/cm^3^)
1	1.8	0.6	0.804
2	1.8	0.8	1.072
3	1.8	1.0	1.340
4	5.0	0.6	1.072
5	5.0	0.8	1.340
6	5.0	1.0	0.804
7	14.7	0.6	1.340
8	14.7	0.8	0.804
9	14.7	1.0	1.072

**Table 2:** Taguchi orthogonal matrix for the DOE2, where *L* indicates the PLGA chain length, *H* the hydration level, and *P* the PLGA density

	*L* (nm)	*H* (g/cm^3^)	*P* (g/cm^3^)
1	1.8	0.0	0.804
2	1.8	0.3	0.804
3	5.0	0.0	1.072
4	5.0	0.3	1.072
5	5.0	0.6	1.072
6	14.7	0.0	1.340
7	14.7	0.3	1.340
8	14.7	0.6	1.340
(9)	1.8	0.6	0.804

Notice that the configuration 9 of DOE2 is the same than the configuration 1 of DOE1

After calculating the *D* of water, PLGA, and curcumin from MD simulations, the response space could be determined. The goal was to find a correlation representing the response trends across the range of design variables. A multiple linear regression model was initially considered for fitting MD results:

2 $$\begin{aligned} \ln (D)=C+A_1 \cdot L+A_2 \cdot H + A_3\cdot P , \end{aligned}$$

being $A_i$ and *C* fitting coefficients. A logarithmic transformation was adopted for *D* in Eq. 2, given the various orders of magnitude explored by the different diffusion mechanisms. Subsequently, a more sophisticated multiple linear regression model with interaction terms was explored in the form of:

3 $$\begin{aligned} \ln (D)= & {} C+A_1 \cdot L+A_2 \cdot H+A_3 \cdot P\nonumber \\{} & {} +B_1\cdot L \cdot H+B_2 \cdot P \cdot L +B_3 \cdot P \cdot H , \end{aligned}$$

being $A_i$, $B_i$, and *C* fitting coefficients. Notice that the regression analysis was performed considering the following measurement units: $[D]= \mu$m^2^/s, $[L]=$ nm, $[H]=$ g/cm^3^, and $[P]=$ g/cm^3^. To avoid over-fitting, no further increase in the model degree was considered.

**Fig. 3:**
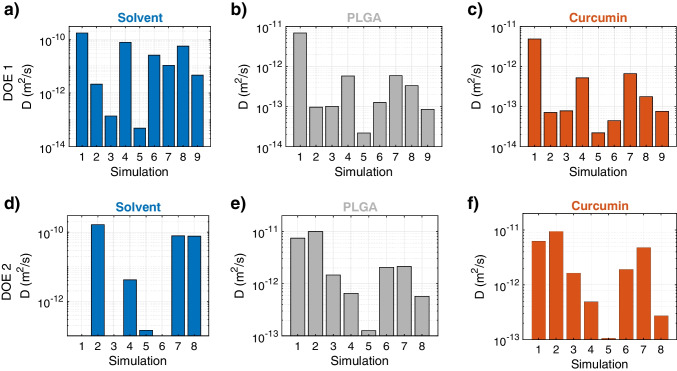
Histograms of diffusion coefficient (*D*) for water (**a** and **d**), PLGA (**b** and **e**), and curcumin (**c** and **f**) for DOE1 and DOE2, respectively — logarithmic scale

## Results and discussion

### Molecular dynamics results

As a reference, we initially tested a single curcumin in a water box (cubic domain with 10 nm side; $T=$ 300 K and $p=$ 1 bar) observing $D=1.0\times 10^{-10}$ m^2^/s for the curcumin, showing a box size-independent value. This result is in the same order of magnitude of previous data in the literature: for instance, Ilnytskyi et al. [58] reported $D=4.0\times 10^{-10}$ m^2^/s for a single curcumin molecule in water, being the difference ascribable to the smaller computational domain and the coarser OPLS-UA (united atoms) force-field employed in this previous work.

In Fig. 3, we present the calculated diffusion coefficients for both DOE1 and DOE2 configurations in logarithmic scale, categorized by the three species present in the system (see Supplementary Tables S1 and S2 for tabulated results). The reported *D* values were calculated from stable MSD (see Supplementary Note 3 and Supplementary Figs. S1, S2, S3, S4, S5, and S6), thus assuring steady-state measures. In general, as observed in both DOE1 and DOE2, the diffusivity of water is higher than that of the other two materials (see Fig. 3a and d), due to its lower molecular weight [59, 60]. The average self-diffusion coefficient of water in the different configurations tested is found to be $D=5.2 \times 10^{-11}$ m^2^/s, being sensibly lower than its bulk value at ambient conditions ($2.4-2.7 \times 10^{-9}$ m^2^/s [61]). This evidence demonstrates a nanoconfined state of water and thus reduced mobility, coherently with previous observations in the literature [62–67]. As evident in Fig. 3a and d, the degree of water nanoconfinement and thus *D* value is strongly related with the tested configuration, with different sensitivities with respect to *L*, *H*, and *P* parameters.

Results in Fig. 3c and f show that the *D* of curcumin through the hydrated PLGA is significantly lower as compared with that of curcumin in water alone, because of the enhanced diffusion resistance induced by PLGA chains. The obtained curcumin diffusion coefficients lie in a range between $D=2.2\times 10^{-14}$ m^2^/s (configuration 5, DOE1) and $9.4\times 10^{-12}$ m^2^/s (configuration 2, DOE2), obtaining comparable values with respect to previous results in the literature analyzing similar — but not identical — setups [58, 68–70]. In detail, Samanta and Roccatano [68] performed MD simulations of curcumin and pluronic block copolymer in different liquid environments, observing a value of diffusion coefficient of curcumin as low as $D=6.0\times 10^{-11}$ m^2^/s. This value is slightly higher than our obtained interval, likely due to the fact that the system and the boundary conditions simulated are different. Karataş et al. [69] carried out density functional theory (DFT) and MD simulations of curcumin and PLGA, obtaining curcumin diffusion values up to $4.6\times 10^{-13}$ m^2^/s depending on the simulated configuration. This value lies in our range, confirming the routine adopted and the reliability of the results obtained. Furthermore, Burin and colleagues [70] showed effective diffusion coefficients of molecules — with comparable molecular weight with respect to curcumin — through PLGA in the order of $10^{-13}$ m^2^/s.

Comparing the diffusion coefficients of curcumin and PLGA in Fig. 3b, c, e, and f, a similar pattern and thus sensitivity can be noticed with respect to *L*, *H*, and *P* parameters. The comparable *D* values for both curcumin and PLGA are due to their similar molecular weight, at least for the relatively short PLGA chains simulated here. Furthermore, higher PLGA and curcumin diffusivities are generally noticed in configurations with lower water nanoconfinement (i.e., higher *D* in Fig. 3a and d), being water viscosity lower in such conditions [71]. Interestingly, the diffusivity of both curcumin and PLGA measured in the DOE1 show a limited variability of values around $D\sim 10^{-13}$ m^2^/s for the different combinations of *L*, *H*, and *P* parameters except for configuration 1, which presents higher $D\sim 10^{-11}$ m^2^/s instead (see Fig. 3b and c). This can be attributed to the fact that the configuration 1 has the lowest level of hydration, PLGA density, and length among the considered computational domains of DOE1, which all lead to a reduced steric occupation and thus diffusion resistance for the curcumin and PLGA motion. Such evidence is corroborated by the higher diffusivities of curcumin in the order of $D\sim 10^{-12}$ m^2^/s more frequently observed in the DOE2, where lower hydration levels are explored. Therefore, an inverse correlation between *D* and *H* can be clearly noticed, while more quantitative regression analyses are required to disentangle the effects of *L* and *P* on diffusivity as well.

**Fig. 4:**
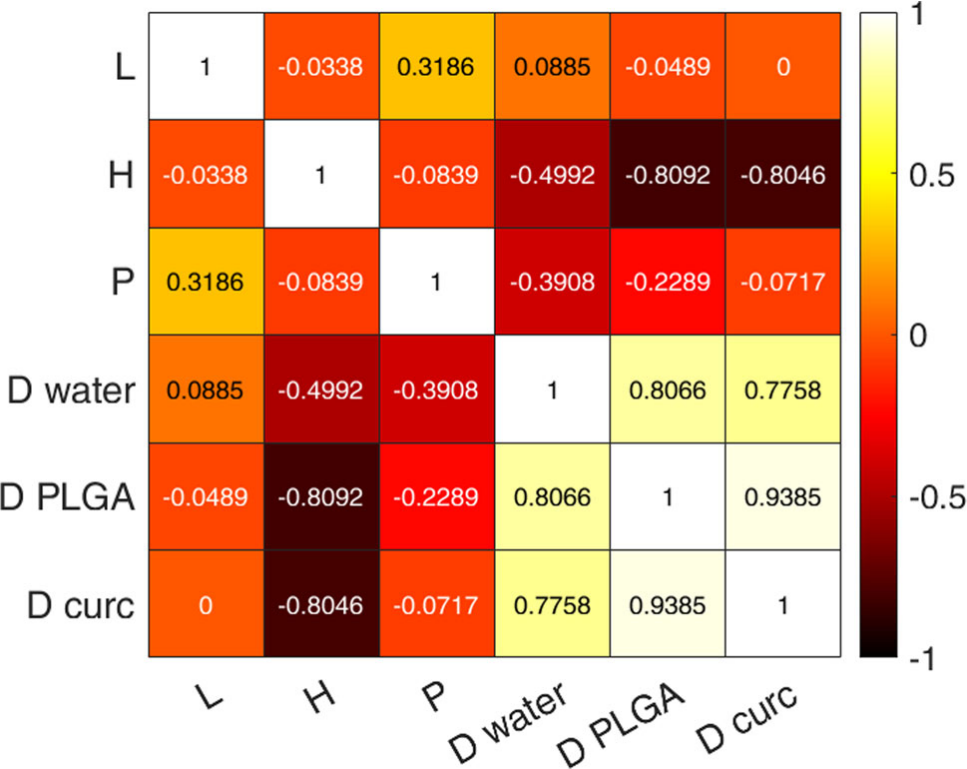
Spearman’s correlation coefficient (from -1 to +1, see color bar) between the input variables (hydration level *H*, PLGA chain length *L*, and PLGA density *P*) and the diffusion coefficients of water, PLGA, and curcumin molecules measured by MD. Levels with $H=0$ g/cm^3^ are not considered in this analysis, to avoid null values of water diffusivity

Such qualitative correlation evidence is systematically analyzed by computing the Spearman’s correlation coefficient between the considered input variables and the obtained diffusivities (see Fig. 4). The analysis confirms a strong positive correlation between the diffusion coefficients of PLGA and curcumin but also highlights their positive correlation with water self-diffusivity. In contrast, *H* is shown to be strongly inversely correlated with the diffusivity of curcumin and PLGA, while a milder correlation is observed with that of water. A more moderate inverse correlation is also visible between *P* and the molecule’s diffusivities, while *L* appears to have a negligible impact on them.

### Regression and sensitivity analysis

A regression was then carried out on the complete set of MD results, considering both DOE1 and DOE2, to understand the sensitivity of each input parameters (*L*, *H*, and *P*) on the diffusion coefficient of curcumin ($D_c$ from now on), which is the key parameter to design drug delivery systems made of PLGA. Considering Eq. 2 as fitting model, the best regression ($R^2_{adj}=0.70$) is achieved with:

4 $$\begin{aligned} \ln (D_c)=4.957-0.001 \cdot L-4.363 \cdot H -3.075 \cdot P. \end{aligned}$$

**Fig. 5:**
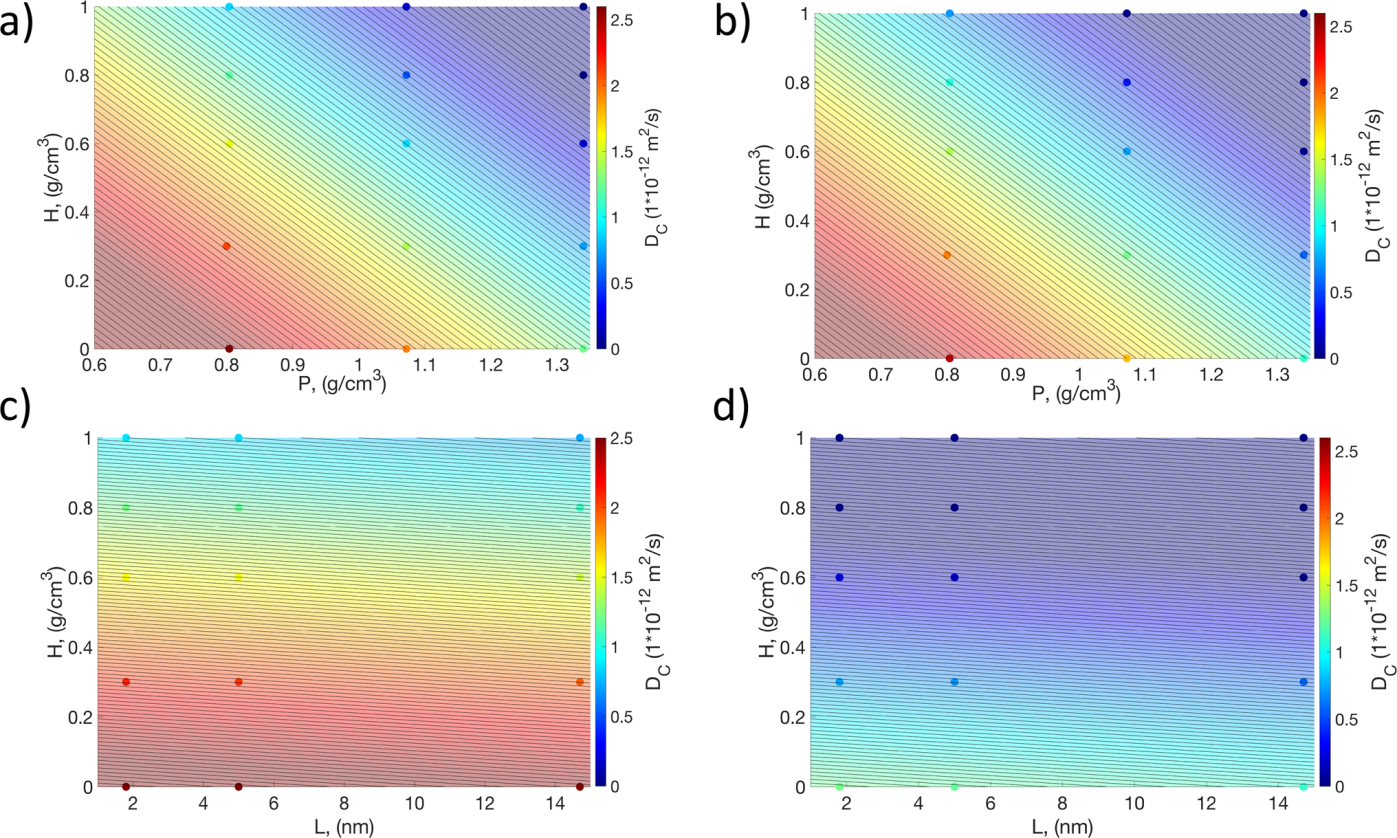
Diffusion coefficient of curcumin ($D_c$) as a function of the hydration level (*H*), PLGA chain length (*L*), and PLGA density (*P*) explored by MD in this study (dots), and best fitted by Eq. 4 (background, continuous color). **a** $L=$ 1.8 nm. **b** $L=$ 14.7 nm. **c** $P=$ 0.804 g/cm^3^. **d** $P=$ 1.340 g/cm^3^

To visualize Eq. 4 in the range of input parameters explored in this work, a 2D color map is reported in Fig. 5, where each dot represents one of the simulated configurations. In the first two panels, where the PLGA length is kept fixed to the minimum (Fig. 5a) and to the maximum (Fig. 5b) value tested, we observe diagonal contour plots, revealing a comparable influence of PLGA density and hydration level on $D_c$. Moreover, the difference between Fig. 5a and b is minimal, thus indicating the marginal role of PLGA length — at least in the considered range. On the other hand, going from the minimum (Fig. 5c) to the maximum (Fig. 5d) value of PLGA density, the color distribution changes significantly, demonstrating a key role of PLGA density in determining $D_c$. Additionally, as previously noted, lower hydration levels lead to increasing diffusion coefficients; whereas, no significant changes of $D_c$ with PLGA length are evident again.

**Fig. 6:**
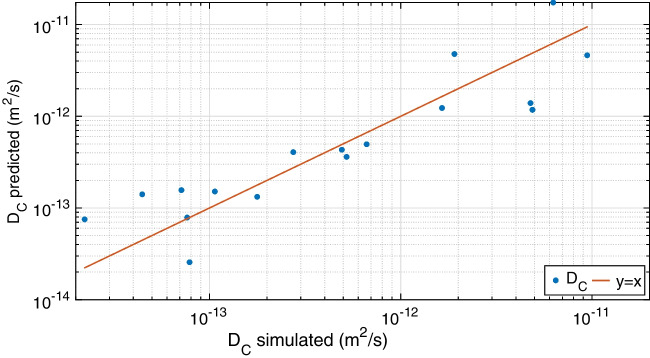
Comparison between the diffusion coefficient of curcumin ($D_c$) calculated with MD simulations and predicted by regression Eq. 6. Points lying on the identity line correspond to the highest regression accuracy

To better quantify and compare the sensitivity of $D_c$ with the different input parameters, standardized regression coefficients were computed as [72]:

5 $$\begin{aligned} A_{i,STD}=A_{i}\frac{S_{X_i}}{S_{Y}} , \end{aligned}$$

where $A_i$ are the best-fitted regression coefficients in Eq. 4, $S_{X_i}$ the standard deviations of the related input data, and $S_Y$ the standard deviation of the output. The standardized regression coefficients delineate the extent to which a dependent variable changes for each increase in the standard deviation of the independent variable.

In our case, we found that the standardized coefficient of PLGA length is $-0.004$, while for hydration level is $-0.787$, and finally for PLGA density is $-0.357$. This means that the hydration level and PLGA density hold the most substantial influence on $D_c$ through an inverse proportionality, whereas the impact of the PLGA length can be considered negligible in comparison to the other two parameters. In detail, the negative values of the standardized coefficients in the linear fit equation for *H* and *L* corroborate that — at a given box size — an increasing number of water and PLGA molecules is related to higher steric hindrance and reduced free volume available for curcumin diffusion. We remark that the simulated range of *L* was limited by the computational feasibility of the simulated domain; however, the longer chains typically available experimentally could also lead to increased entanglement and slower mobility of the polymer matrix, consequently causing a different sensibility of $D_c$ on *L*, which should therefore investigated further via, e.g., mesoscopic simulations.

Finally, the linear regression model with interaction terms in Eq. 3 was best fitted to obtain an improved prediction accuracy ($R^2_{adj}=0.78$), that is:

6 $$\begin{aligned} \ln (D_c)= & {} 8.205-0.422\cdot L-6.014\cdot H -6.447\cdot P \\{} & {} - 0.01 \cdot L \cdot H+0.410 \cdot P \cdot L+1.918 \cdot P \cdot H .\nonumber \end{aligned}$$

The enhanced interpolation of MD results for $D_c$ can be appreciated also in Fig. 6, where the computed values from numerical simulations are compared with the predicted values from Eq. 6. In perspective, Eq. 6 could be adopted to link molecular simulations with continuum approaches in multi-scale models of curcumin diffusion and release from PLGA-based carriers, where further effects like advection, thermophoresis, particle swelling, or degradation should be considered as well.

## Conclusions

Nano- and micro-technology can be a powerful tool for synthesizing and improving drug delivery systems, targeting specific sites in the body without damaging healthy tissues and cells. Additionally, tailored drug delivery systems help to release and monitor the drug dose over a specific time interval, becoming independent of cumbersome machinery and medical routines that debilitate daily activities [73]. Nevertheless, there is a lack of understanding that can link the drug diffusion process to the design parameters of the delivery system. For this reason, it is urgent to investigate and quantify the diffusion processes that physically govern the release of drugs from carriers with molecular precision. The use of PLGA as the carrier is among the most studied and promising materials for drug delivery systems, thanks to its superior biodegradability and biocompatibility characteristics. On the other hand, curcumin has promising anti-inflammatory, and anticancer properties. Here, an *in silico* study investigated a drug delivery system composed of curcumin encapsulated in a hydrated PLGA matrix at the molecular scale.

First, the topology of the representative volume element of the system was built, considering PLGA, curcumin, and water molecules as building blocks. Afterward, molecular dynamics simulations were performed to calculate the MSD of the species at a steady state. These results were found to be stable over time, hence the diffusion coefficients were extracted using the Einstein formula for Brownian motion. The latter were found to agree with the few data found in the literature, confirming the goodness of the chosen models and the adopted routine for the simulation.

MD results showed that curcumin and PLGA have comparable values of diffusion coefficients for the considered case studies, while water has higher diffusivity due to its lighter molecular weight. The sensitivity analysis showed that the hydration level and PLGA density play the most significant role in influencing the diffusion mechanism of curcumin, while the length of the PLGA chain is negligible compared to the other two — at least for the simulated range of values. Additionally, the coefficients of hydration level and PLGA density in the linear regression equation indicate that an increase in any of these two input parameters corresponds to a decrease in the diffusion coefficient of curcumin, this being due to the steric hindrance to curcumin diffusion. In summary, this computational approach has revealed interesting relationships between the considered input parameters and the diffusion coefficient, providing an accurate regression model to quantify them.

Overall this work represents the first step of a bottom-up multi-scale approach proposing a rational design process to assist the development of drug delivery systems such as Discoidal Polymeric Particles. Predicting the diffusion processes of drug nanoparticles into the carrier PLGA matrix depending on different material parameters and molecular configurations is a key aspect to help in the development of drug delivery particles with targeted properties. Finally, we believe that this approach can be efficiently adopted for future studies and seamlessly integrated with approaches at larger scales (e.g., finite element method).

### Supplementary Information

Below is the link to the electronic supplementary material.

Supplementary file 1 (pdf 2123 KB)

## Data Availability

All data generated or analyzed during this study are included in this published article and the supplementary material file.
