# Supplementary material for: Diffusion of curcumin in PLGA-based carriers for drug delivery: a molecular dynamics study
Source: J Mol Model. 2024 Jun 19;30(7):219. doi: 10.1007/s00894-024-06023-x (PMC11186890; doi:10.1007/s00894-024-06023-x)
Supplement: Supplementary file 1 — (pdf 2123 KB) [file 894_2024_6023_MOESM1_ESM.pdf]

Diffusion of curcumin in PLGA-based carriers for drug delivery: A molecular dynamics study – Supplementary Material –

Alessandro De Giorgi,† Francesco Bellussi,† Stefano Parlani,† Andrea Lucisano,† Emanuele Silvestri,† Susmita Aryal,‡ Sanghyo Park,‡ Jaehong Key,*,‡ and Matteo Fasano*,†

†*Department of Energy, Politecnico di Torino, Torino, Italy*

‡*Department of Biomedical Engineering, Yonsei University, Wonju, Republic of Korea*

E-mail: jkey@yonsei.ac.kr; matteo.fasano@polito.it

In this document we provide supplementary notes, tables and figures with additional details on model development and obtained results.

Supplementary Notes

Supplementary Note 1: Tukey's method

Tukey's method is based on measures such as the interquartile range, where the lower and the upper quartiles of a series of measurements are used as range to detect as an outlier any observation outside it. A mathematical formulation for the range is:

$$[Q_1 - k(Q_2 - Q_1), Q_2 + k(Q_2 - Q_1)], \tag{S1}$$

where Q_1 and Q_2 are the lower and upper quartiles, respectively, and k is a constant which is equal to 1.5 in the Tukey's method.[1] Thus, when an outlier value for D was found during a MD trajectory, the simulation to which it belonged was run again, in order to get rid of it.

Supplementary Note 2: Design of Experiment

A Design of Experiment (DOE) is a statistical approach that differs from a traditional experimental method. The latter performs a loop such as developing a theoretical hypothesis, performing a trial to confirm such hypothesis, analyzing results, finding corrections to make, and performing further experiments until a good result is reached. Such a method can result in a long and not optimized process and furthermore it requires both a complete theoretical knowledge of the studied factors and the capability to control them. A DOE mitigates this disadvantages and it is more adequate to this study case, since the diffusion mechanism of curcumin in PLGA was relatively unknown and dependent on numerous variables. The chosen structure of the DOE is the so called Taguchi Method.

Taguchi Method (TM) is an alternative to a full-factorial method (FFM), because both take into account the possibility of interactions among parameters considering that the model sensitivity to one parameter can change depending on the values of other parameters. A FFM analysis generally requires a large number of experiments, which is impractical for time

consuming simulations such as MD ones. Furthermore, the expected experiments result to be often redundant, adding little or even no new information. Hence, TM results particularly adapt to this case, since it does not require too many trials. An orthogonal array (OA) is used to reduce the number of simulations and obtain reasonable information.[2] Writing the orthogonal array matrix involves a procedure also known as the *fractional factorial design* technique.

In the considered domain, the three input variables to be explored are: the hydration level, the PLGA length, and density. Considering these three variables and three levels per each one of them, the TM required a number of trials equal to 3^2. Hence a L9 matrix was designed, meaning that the design space is composed by three values for each sample and thus nine possible configurations to be simulated.

After the DOE structure and the orthogonal matrix have been written, the next step is to conduct the matrix experiment. The following protocol was followed to build the target MD configurations:

1. Placing the curcumin in the center of a sphere formed by packed PLGA chains with a given length.

2. Loading the packed ensemble in an opportunely large cubic box.

3. Performing an energy minimization of the system to relax the compacted chains.

4. Introducing a number of water molecules in the computational box needed to reach the target hydration level, performing a further energy minimization after the solvation process. The water density was computed with respect to the solvent accessible volume in the box.

5. Performing a series of NVT and NPT simulations to equilibrate the temperature and pressure of the system and reach the target PLGA density, while adjusting the number of water molecules with the updated solvent accessible volume in the box. An accept-

able margin of error on the final PLGA density was established to be ±5% with respect to the target values.

The final box size, number of water molecules and number of total atoms for DOE1 and DOE2 are summarized in Tabs. S3 and S4. After performing this preparation procedure for all configurations prescribed by TM, NVT production runs are carried out to generate statistically relevant trajectories (5 ns) and finally to post-process the diffusivities of water, PLGA, and curcumin molecules.

Supplementary Note 3: Simulation convergence

The time evolution of the MSD for all the simulations of DOE1 and DOE2 are presented in Figs. S1, S2, S3, S4, S5 and S6. Each D value was calculated from the linear fitting of these MSD trajectories, following the Einstein relation. As it can be noticed from Figs. S1, S2, S3, S4, S5 and S6, all simulations are stable, with diffusivity values that pass the Tukey's method check, proving the convergence of results.

Additionally, a test simulation (*i.e.*, sim 7 of DOE1) was conducted for up to 10 ns to check the system's convergence. As shown in Fig. S7, the mean squared displacement (MSD) of each species remains stable over time. We calculated the diffusion coefficients for this extended simulation, obtaining the following results: for water, $D = 11.176 \cdot 10^{-12} m^2/s$; for curcumin, $D = 0.649 \cdot 10^{-12} m^2/s$; and for PLGA, $D = 0.592 \cdot 10^{-12} m^2/s$. The percentage relative errors between the 10 ns and 5 ns simulations were then computed for each species, yielding a discrepancy of 4.82% for water, 2.14% for curcumin, and 0.42% for PLGA. Thus, we considered the differences between the diffusion coefficients to be negligible and regarded all the 5 ns simulations as sufficiently long to guarantee convergence. The resulting diffusion coefficients and the linear fitting interval times for each simulation are summarized in Tabs. S1 and S2 for DOE1 and DOE2, respectively.

Supplementary Tables

Table S1: Diffusion coefficients and linear fitting interval time for DOE1.

	Curcumin		PLGA		Water	
**Sim**	D_C [10^{-12} m^2/s]	**Linear fitting time interval [ps]**	D [10^{-12} m^2/s]	**Linear fitting time interval [ps]**	D [10^{-12} m^2/s]	**Linear fitting time interval [ps]**
**1**	4.882	50-4000	6.868	50-4000	171.270	50-5000
**2**	0.071	50-5000	0.096	50-5000	2.126	50-5000
**3**	0.079	50-5000	0.102	50-5000	0.139	50-5000
**4**	0.521	50-5000	0.582	50-5000	77.225	50-4000
**5**	0.022	50-5000	0.022	50-4000	0.049	50-4000
**6**	0.045	50-5000	0.127	50-5000	25.460	50-5000
**7**	0.663	50-5000	0.590	50-5000	10.663	50-5000
**8**	0.178	50-5000	0.333	50-5000	55.770	50-5000
**9**	0.076	50-5000	0.086	50-4000	4.613	50-5000

Table S2: Diffusion coefficients and linear fitting interval time for DOE2. N.A. refers to Not Applicable, when water molecules are not included in the simulation.

	Curcumin		PLGA		Water	
**Sim**	D_C [10^{-12} m^2/s]	**Linear fitting time interval [ps]**	D [10^{-12} m^2/s]	**Linear fitting time interval [ps]**	D [10^{-12} m^2/s]	**Linear fitting time interval [ps]**
**1**	6.270	50-5000	7.422	50-5000	N.A.	N.A.
**2**	9.436	50-4000	9.972	50-4000	165.250	50-5000
**3**	1.644	50-5000	1.469	50-4000	N.A.	N.A.
**4**	0.492	50-4000	0.652	50-4000	4.165	50-5000
**5**	0.107	50-1000	0.126	50-1000	0.145	50-4000
**6**	1.901	675-4000	2.054	50-4000	N.A.	N.A.
**7**	4.770	50-1000	2.135	50-5000	78.371	50-5000
**8**	0.274	2600-5000	0.571	50-5000	76.922	50-4000

Table S3: Box size, number of water molecules and total number of atoms for DOE1.

Sim	Box size (x = y = z) [nm]	Water molecules	Number of atoms
**1**	2.87657	477	2843
**2**	2.64874	497	2903
**3**	2.45745	497	2903
**4**	3.54251	892	6197
**5**	3.32699	986	6470
**6**	4.01852	2170	10022
**7**	5.66354	3650	27557
**8**	6.33239	6800	37007
**9**	6.15751	7810	40037

Table S4: Box size, number of water molecules and total number of atoms for DOE2.

Sim	Box size (x = y = z) [nm]	Water molecules	Number of atoms
**1**	2.87567	0	1412
**2**	2.87567	239	2129
**3**	3.32699	0	3512
**4**	3.32699	370	4622
**5**	3.32699	739	5729
**6**	6.10046	0	16607
**7**	6.10046	2280	23447
**8**	6.10046	4560	30287

Supplementary Figures

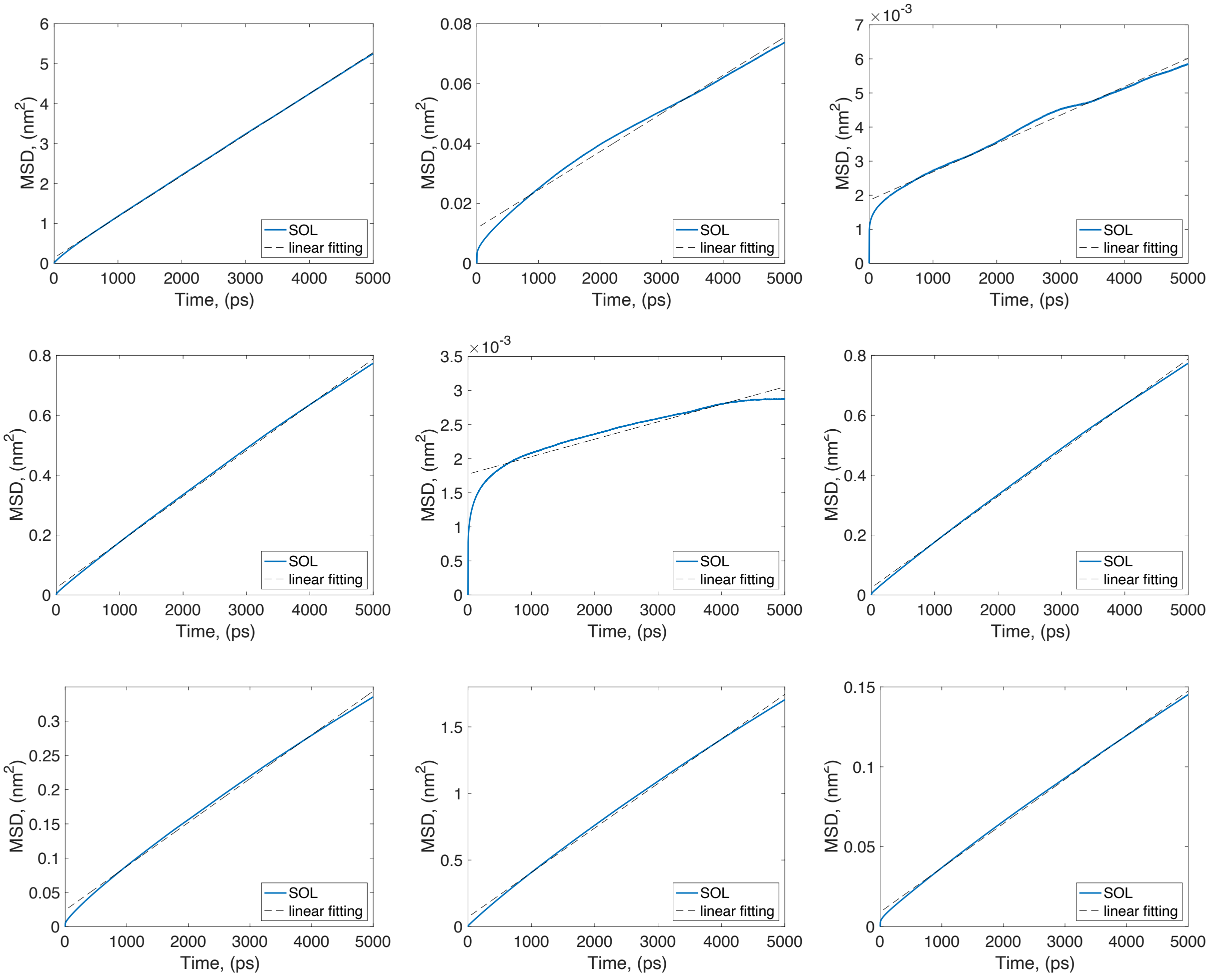

Figure S1: Time evolution of solvent MSD in DOE1 with linear fitting for D calculation, from configuration 1 to 9 (from left to right; from top to bottom).

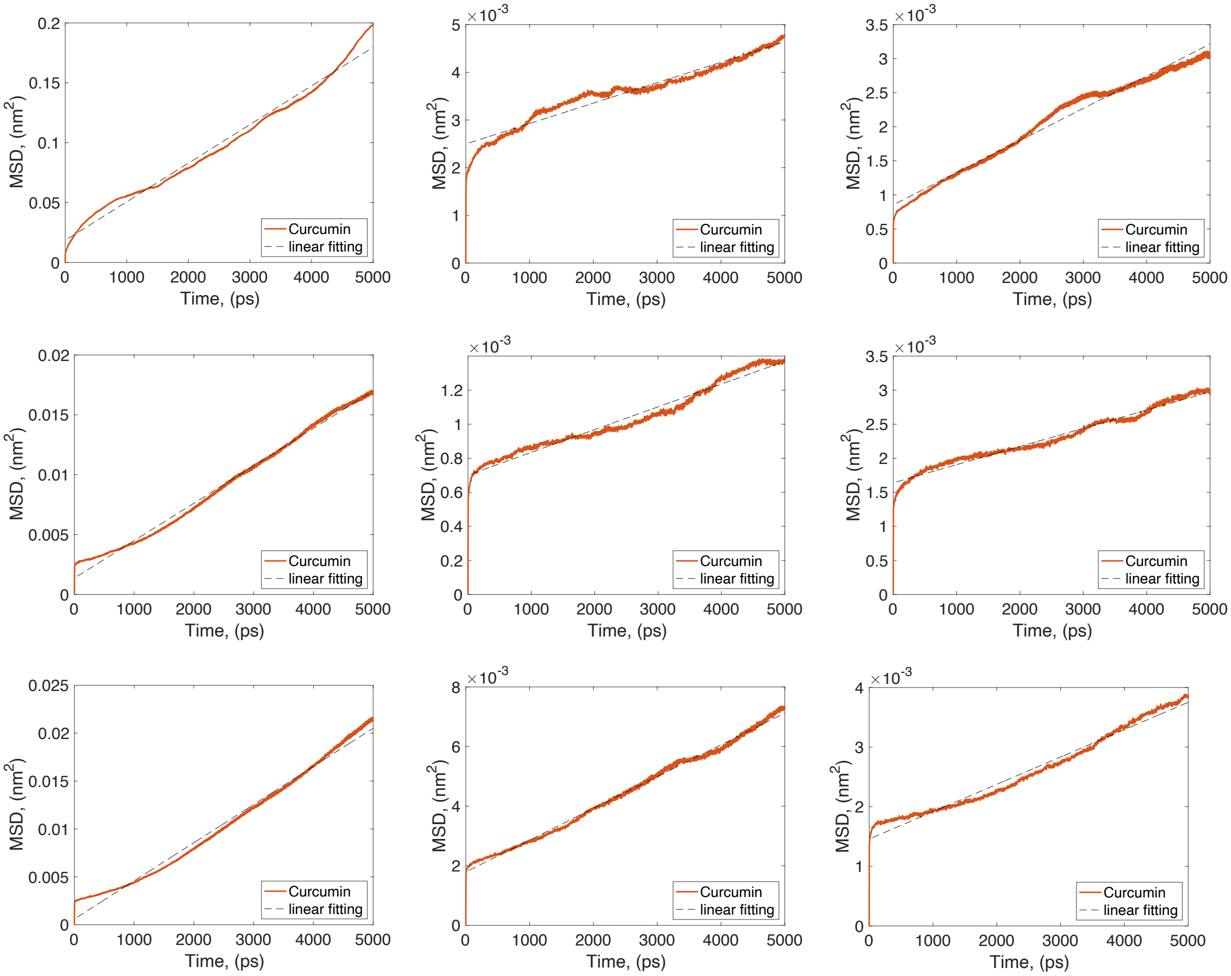

Figure S2: Time evolution of curcumin MSD in DOE1 with linear fitting for D calculation, from configuration 1 to 9 (from left to right; from top to bottom).

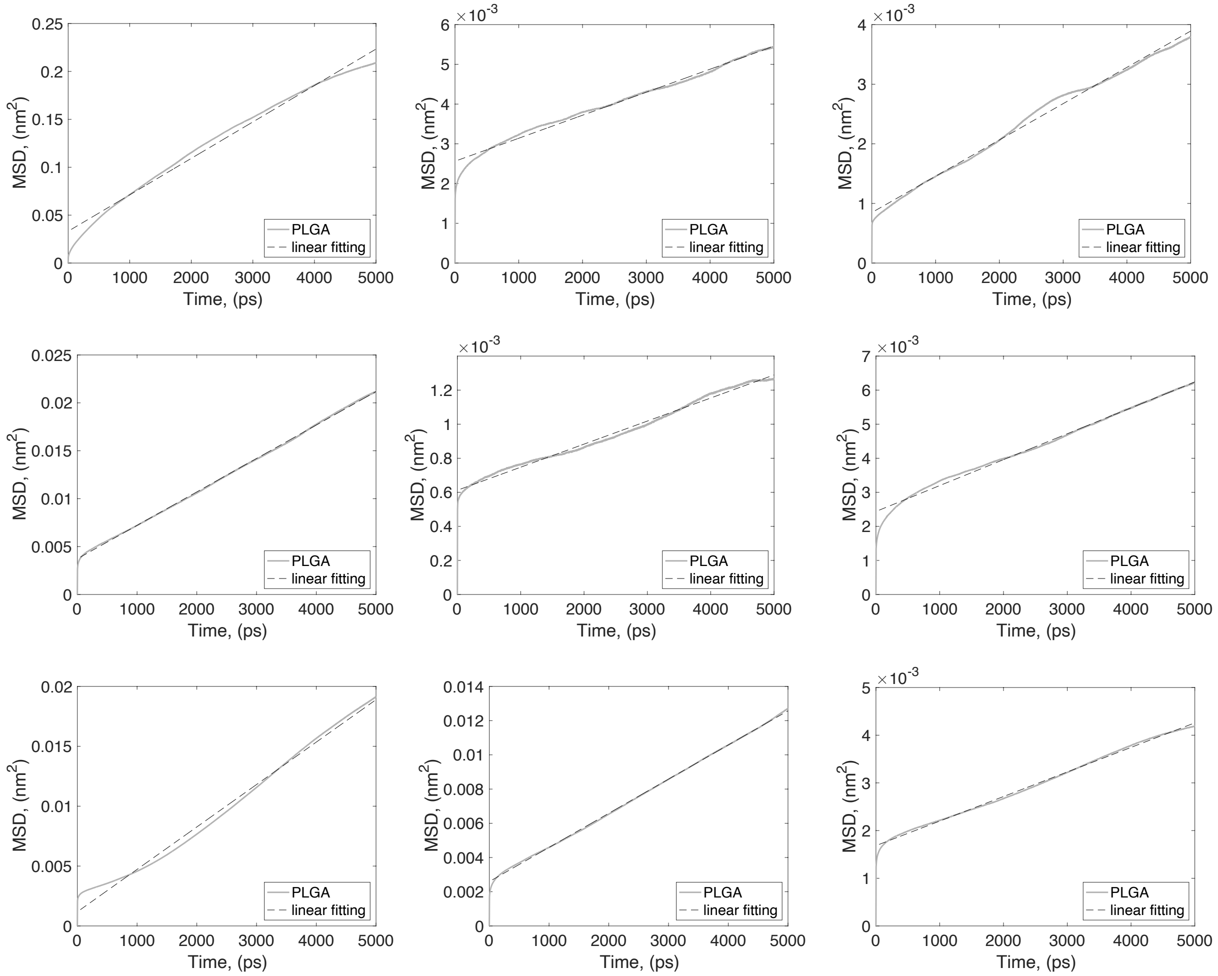

Figure S3: Time evolution of PLGA MSD in DOE1 with linear fitting for D calculation, from configuration 1 to 9 (from left to right; from top to bottom).

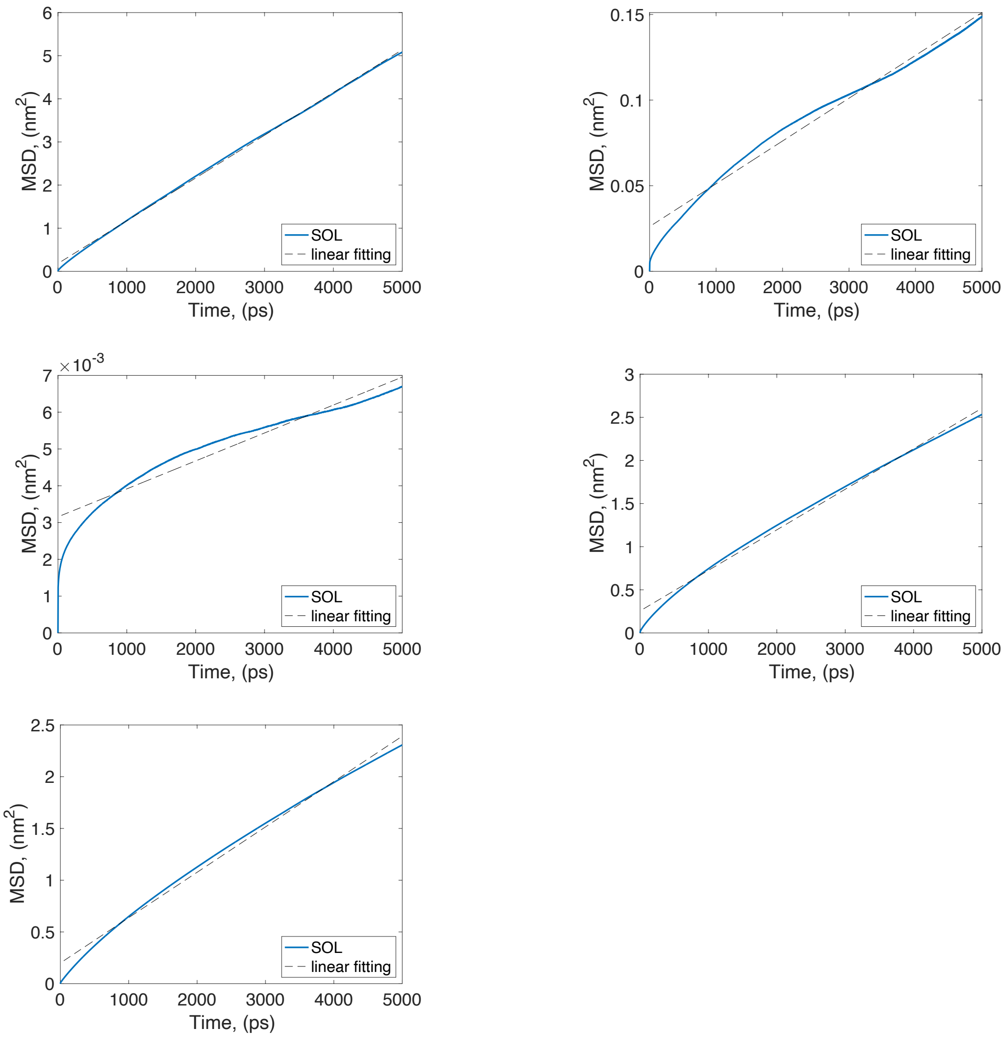

Figure S4: Time evolution of solvent MSD in DOE2 with linear fitting for D calculation, from configuration 2 to 8, ignoring 3 and 6 in which there is no solvent (from left to right; from top to bottom).

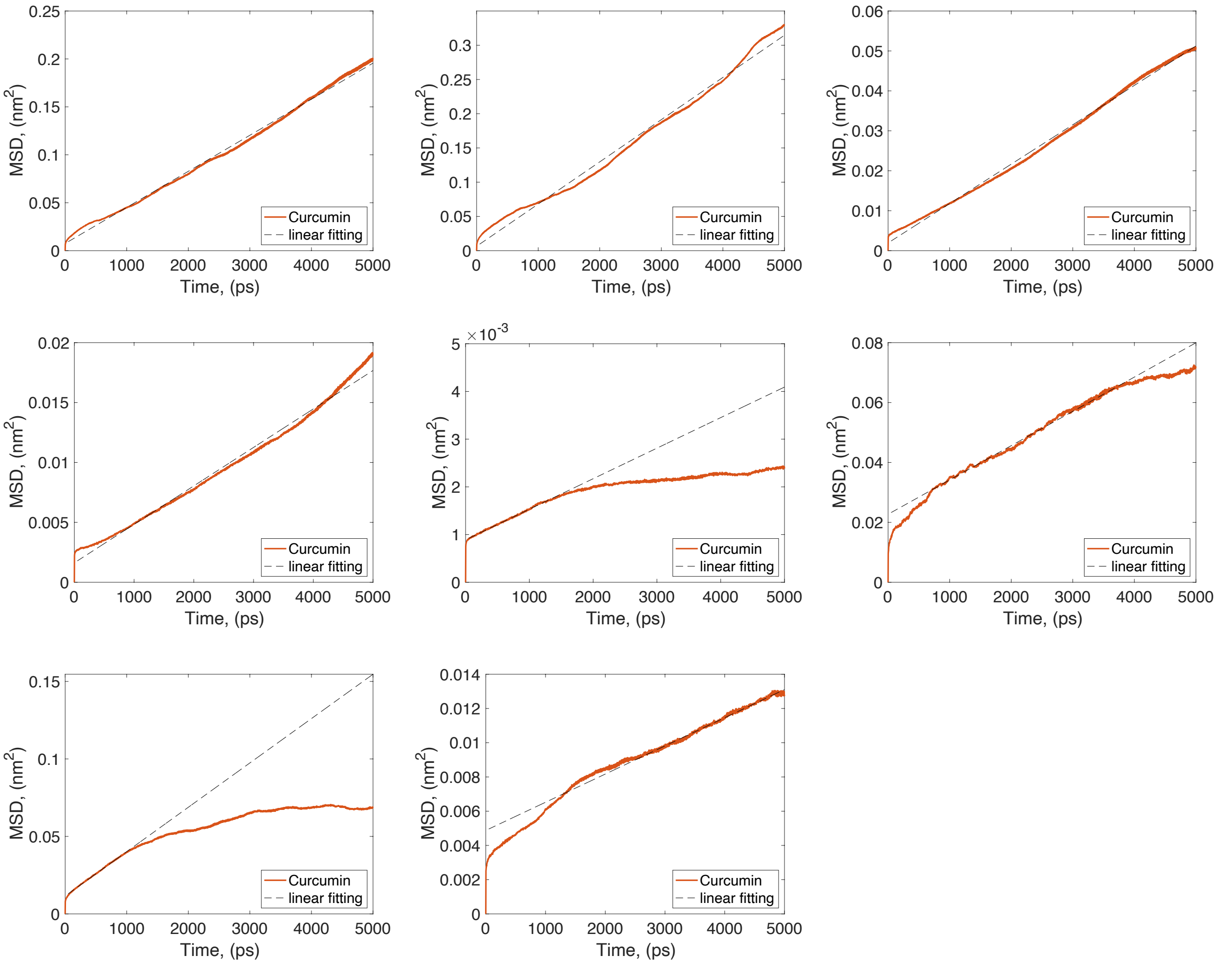

Figure S5: Time evolution of curcumin MSD in DOE2 with linear fitting for D calculation, from configuration 1 to 8, (from left to right; from top to bottom).

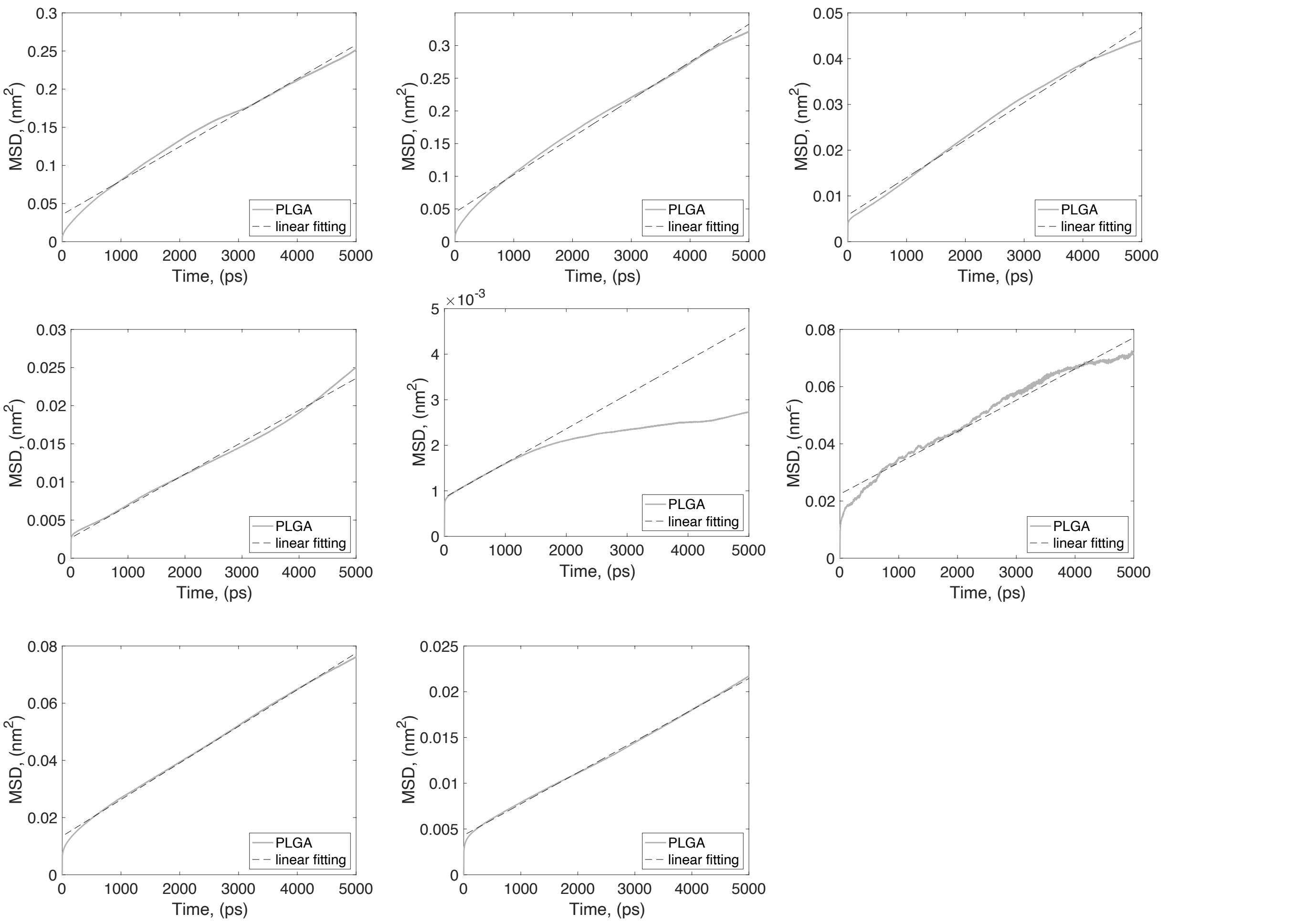

Figure S6: Time evolution of PLGA MSD in DOE2 with linear fitting for D calculation, from configuration 1 to 8, (from left to right; from top to bottom).

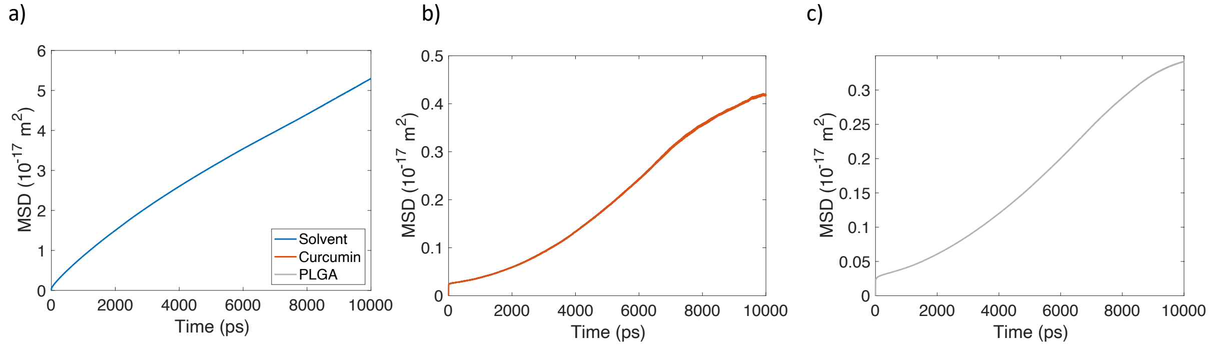

Figure S7: 10 ns time evolution of MSD in a simulation test (DOE1, sim7).

References

(1) Seo, S. A review and comparison of methods for detecting outliers in univariate data sets. *BS, Kyunghee University* **2002**,

(2) Qi, L.; Mikhael, C. S.; Funnell, W. R. J. Application of the Taguchi method to sensitivity analysis of a middle-ear finite-element model. **2004**,
